# A Synopsis of Systematic Botany

**Published:** 1833-10

**Authors:** 


*A Synopsis of Systematic Botany, as connected with the Plants admitted into the Pharmacopœias of London, Edinburgh, and Dublin; accompanied by a Planisphere, shewing at one View the Class and Order of the Medical Genera, according to Linnæus and Jussieu.* By Thomas Castle, F.L.S. &c. London, 1833. 4to. pp. 17.

It is the opinion of a celebrated German physician that anthelmintics are unnecessary medicines; for, says our learned Saxon brother, those parasitic animals we call tæniæ, lumbrici, or ascarides, can exist only when the animal economy is in a low, degraded condition: restore the unfortunate patient to decent average health, give fresh tone to his muscles, and the long-lost elasticity to his step, and, depend upon it, he will be able to eject these loathsome tenants of his bowels, without gamboge, turpentine, or Aspidium filix mas. Now, it seems to us that this dictum of the quaint and original Hahnemann was written for your instruction, O Court of Examiners of the Society of Apothecaries! When you apply to parliament for a new act, demand no clause enabling you to banish *grinders*, or to burn their books; but reform your examinations, make them more healthy, more solid, and the grinder—for man is but a worm—will drop off spontaneously. The strange looking tables of contents which grinders love to palm off as books, must unquestionably be considered as quiet unanswerable sarcasms on the examinations to which they are intended to be passports. Pope or Garth might have written more polished satires, but we doubt if they could have been keener; and the bold botanical examiner who could bear the heavy blows of the "Synopsis" without flinching, might endure the knout with the most courtly serenity. But we do not expect any examiner to display this unwincing apathy. The *ironie sanglante* of Mr. Castle cannot be misunderstood: he says, as plainly as a "planisphere" can speak, "Your examinations turn on routine phrases, and dry classification, the mere skeleton of botany; a good crammer might inject a passing knowledge of all this into a pupil in two hours. Reform it altogether. Put a dozen plants on the table, selected not from a hundred medical ones, but from the boundless stores of the Hortus Britannicus; ask the young tyro to describe these; question him as to their natural affinities, and probable properties; observe, too, if he possesses the invaluable art of scientific analysis, and can thus reduce the number of classes to which an unknown object may possibly belong. If he can do this, or only a part of it, (for we do not expect to find a Burnett in every tyro,) he is on the road to knowledge, and incomparably superior to one who can say the *Synopsis* by heart, without missing a word."

We are afraid that Mr. Castle will complain that we have dealt out but scanty justice to him, in stating that this Synopsis contains only seventeen pages; for, in reality, the eighteenth page, though not numbered, is covered with advertisements of Mr. Castle's works. They are eight in number, of which six are born, and two unborn. Four have a planisphere, four have none. The advertisement of one of the "planispheric" books has given us so much pleasure, that it would be selfish to deny our readers a participation in it; and we shall therefore quote the whole, with the seal of approbation stamped on Mr. C.'s book by a kindred genius.

"V. Price four shillings and sixpence. A table for finding the commencements, characteristics, and regular inflexions of Greek verbs; by affording at one view: first, a list of the letters which may commence a verb, with the manner in which they respectively receive the augment; secondly, a list of the characteristic letters of the present tense, with the change that takes place in the formation of the four other fundamental tenses; thirdly, a table for finding the person, number, tense, mood, and voice of any inflexion of regular verbs in ω."

"This is one of the neatest and most ingenious pieces of planispheric engraving we have seen. The arrangement is extremely simple, and, any person at all acquainted with the Greek language may easily understand the table."—*Literary Gazette, Jan.* 19*th,* 1833.

Capital! This is evidently a hard hit at some examination, but where held we know not. The potent aristocracy of Blackfriars do not meddle with Greek, and, amid the manifold Greek examinations we have passed through, (a score or thereabouts,) we do not recollect a single one where any benefit could have been derived from tables of the augments, &c. being put into a circular form. For ourselves and our children, we shall be content with books printed in strait lines; but we see clearly that Mr. Castle is acquainted with the derivation and original meaning of the word encyclopædia —*instruction in a circle.*

